# Commercial Off-The-Shelf Video Games for Reducing Stress and Anxiety: Systematic Review

**DOI:** 10.2196/28150

**Published:** 2021-08-16

**Authors:** Federica Pallavicini, Alessandro Pepe, Fabrizia Mantovani

**Affiliations:** 1 Department of Human Sciences for Education “Riccardo Massa” University of Milano Bicocca Milano Italy

**Keywords:** commercial off-the-shelf video games, video games, stress, anxiety, relaxation

## Abstract

**Background:**

Using commercial off-the-shelf video games rather than custom-made computer games could have several advantages for reducing stress and anxiety, including their low cost, advanced graphics, and the possibility to reach millions of individuals worldwide. However, it is important to emphasize that not all commercial video games are equal, and their effects strongly depend on specific characteristics of the games.

**Objective:**

The aim of this systematic review was to describe the literature on the use of commercial off-the-shelf video games for diminishing stress and anxiety, examining the research outcomes along with critical variables related to computer game characteristics (ie, genre, platform, time of play).

**Methods:**

A systematic search of the literature was performed following the PRISMA (Preferred Reporting Items for Systematic Reviews and Meta-Analysis) guidelines. The search databases were PsycINFO, Web of Science, Medline, IEEExplore, and the Cochrane Library. The search string was: [(“video game*”) OR (“computer game*”)] AND [(“stress”) OR (“anxiety”) OR (“relaxation”)] AND [(“study”) OR (“trial”) OR (“training”)].

**Results:**

A total of 28 studies met the inclusion criteria for the publication period 2006-2021. The findings demonstrate the benefit of commercial off-the-shelf video games for reducing stress in children, adults, and older adults. The majority of the retrieved studies recruited young adults, and fewer studies have involved children, middle-aged adults, and older adults. In addition to exergames and casual video games, other genres of commercial off-the-shelf games helped to reduce stress and anxiety.

**Conclusions:**

Efficacy in reducing stress and anxiety has been demonstrated not only for exergames and casual video games but also for other genres such as action games, action-adventure games, and augmented reality games. Various gaming platforms, including consoles, PCs, smartphones, mobile consoles, and virtual reality systems, have been used with positive results. Finally, even single and short sessions of play had benefits in reducing stress and anxiety.

**Trial Registration:**

International Platform of Registered Systematic Review and Meta-analysis Protocols INPLASY202130081; https://inplasy.com/?s=INPLASY202130081

## Introduction

### Background

Since the emergence of the COVID-19 pandemic in 2020, the frequency of stress and anxiety has markedly increased worldwide [1-6], with a prevalence of 29.6% and 31.9%, respectively [6]. The fear of contracting the virus, changes in lifestyle behaviors, social isolation, boredom, and uncertainty have exacerbated stress and anxiety in populations globally, which likely has long-lasting psychological and physical consequences [6-8]. Therefore, finding age-appropriate and cost-effective ways to support individuals in managing stress, anxiety, and their effects is urgently needed [9-11].

Video games represent one of the most appealing technological interventions for developing programs to reduce stress and anxiety since they are motivating, engaging, and easily accessible [12]. In 2020, the number of gamers worldwide was estimated at approximately 2.6 billion, and the games market is expected to exceed US $200 billion by the end of 2023 [13]. In contrast to popular belief, which views male children or teenagers as typical gamers, the average age range of video game players is 35-44 years, and across all players, 59% are male and 41% are female [14].

Computer games go far beyond the boundaries of entertainment. Video games are increasingly being used in sectors such as education [15-17] and mental health [18-21]. Some studies have highlighted the potential dangers of video games in terms of problematic use [22-25] and their relation to psychological functioning [23,26,27], whereas others have emphasized that the enjoyment and intrinsic motivation often associated with computer games make them a valuable and attractive new learning method [15-17] and offer psychological support to people [18-21].

Many schools, from the elementary to university level, adopt video games. Computer games can help to stimulate individuals in all of the transversal competencies collectively defined as “soft skills” (eg, creativity and the ability to deal with problems) and in teaching specific subjects such as mathematics or history [28-30]. Concerning mental health, several studies have demonstrated the usefulness of video games for training cognitive skills, including attentional processes, memory, and cognitive flexibility, especially in the elderly and adults [20,31-33].

Besides serving as useful tools for training cognitive processes [20,31,34], video games may also have a benefit in reducing stress and anxiety [35,36]. Computer games offer various positive emotions-triggering situations [12,18,36,37]. One of the most commonly reported motives for playing modern video games is the pleasure offered by digital games: people look for and are more willing to buy games that elicit positive emotions (eg, happiness and surprise) and enjoyment [38-40]. Like other pleasurable activities, video game playing stimulates dopamine release, a neurotransmitter linked to sensations of pleasure and reward [41].

The fundamental objective of video games is to entertain the player and elicit positive emotions [12,18], which, as stated by the “broaden-and-build” theory [42,43], have positive effects on the psychological well-being of the individual [44-46]. Positive emotions are considered to form the basis for the growth and flourishing self [46], and are especially important to increase subjective well-being [46-48]. Furthermore, video games can elicit the so-called “flow” state [49-51], defined as “the optimal experience when nothing else matters” [52,53], with benefits including increased self-efficacy, a stronger sense of self, and improved overall quality of life [54-56].

Moreover, in many cases, as is true for other entertainment media, video games play a role in distraction from undesirable emotions such as anxiety and stress by providing a temporary diversion from (real-world) adverse events or emotions [19,57-61].

Finally, video games, especially multiplayer games, offer the possibility of establishing a social connection in playing with friends or with people online [62,63]. This fact has become particularly relevant since the COVID-19 pandemic broke out. Gaming for social compensation might mitigate the experienced emotional distress during pandemic-related self-isolation [64,65].

### Commercial Off-The-Shelf Video Games for Relaxation

Most of the studies performed to date on video games for stress and anxiety reduction have focused mainly on custom-made games (ie, games created ad hoc by researchers to educate, train, or change behavior) [66-68]. This type of game is often defined in the literature as a “serious game” [69], as gaming features are used as the primary medium for serious purposes [66]. Several custom-made video games for relaxation integrate biofeedback techniques into the game modes, such as Deep [70,71], Nevermind [72], MindLight [73,74], Dojo [75,76], and StressJam [77]. Furthermore, studies have shown that ad hoc video games could help adults with anxiety better handle emotional and physiological responses to stressors [78] and improve behavioral performance on anxiety-related stress tasks [79].

Interestingly, in addition to custom-made video games, commercial off-the-shelf (COTS) video games have also shown potential application for improving mental health [19], including the reduction of stress and anxiety (eg, [80-82]). 

Using COTS video games rather than video games created ad hoc could have several advantages, including their low cost and ready-to-use format, advanced graphic quality, and the possibility to reach millions of players worldwide. As underlined in a recent paper, COTS games may disrupt health care over the coming decade [19]. Massive corporate funding for COTS games is often much higher than the budgets available to develop custom-made games, making it possible to reach a very high quality of video games in gameplay and user experience [19]. Besides, in contrast to the limited number of players that usually have the opportunity to try a custom-made game, millions of individuals can play a COTS game.

However, it is important to emphasize that not all COTS video games are equal, and their effects strongly depend on the specific characteristics of the game itself, such as its genre [83,84]. A recent systematic review [35] indicated that casual video games (CVGs), characterized by low cognitive loads and generally short time demands such as Tetris and Angry Birds, represent a particularly useful genre for diminishing stress and anxiety. Several other genres of COTS video games appear to be promising for decreasing stress and anxiety in individuals, including exergames [85] or survival horror games [86].

### Research Questions

Within this context, the aim of this systematic review was to describe the literature on the use of COTS video games for decreasing stress and anxiety. The secondary objective was to organize the research with respect to critical variables related to video game characteristics (ie, genre, platform, time of play).

## Methods

### Databases Searched

A systematic search of the literature was performed on March 31, 2021 by two of the authors (FP and AP) following the Preferred Reporting Items for Systematic Reviews and Meta-Analysis (PRISMA) guidelines [87]. The study was preregistered (March 23, 2021) on the International Platform of Registered Systematic Review and Meta-analysis Protocols (INPLASY202130081). The search databases used were PsycINFO, Web of Science, Medline, IEEExplore, and Cochrane Library. 

### Inclusion Criteria

In line with the PRISMA guidelines [87], the authors (FP, AP, FM) established clear inclusion criteria to determine a paper’s eligibility for inclusion in the review. Only studies meeting the following criteria were considered eligible for inclusion: (1) human participants (clinical and nonclinical populations); (2) COTS video games played on a console, mobile console, PC, smartphone/tablet, or virtual reality device; (3) the comparator group was usual care intervention, nonvideo game group, or none; (4) the outcomes measured were levels of stress, anxiety, or both; and (5) the study design was a randomized controlled trial (RCT; ie, participants are randomly assigned to an experimental group or a control group), quasiexperimental (ie, nonequivalent groups, pretest-posttest, and interrupted time series)**,** or cross-sectional/correlational (ie, employing questionnaires and large samples).

Papers published in English in peer-reviewed journals were selected and subjected to a check for the above inclusion criteria. According to the PRISMA guidelines, the authors (FP, AP, FM) established a specific date range; studies published between January 2006 and March 2021 were selected. This period was chosen as the first reports of the effects of video games on stress and anxiety reduction appeared around the 2010s [36].

### Exclusion Criteria

Studies were excluded if they: (1) did not focus on the use of COTS games for diminishing stress and/or anxiety; (2) focused on games that did not meet the definition of a COTS game (ie, “games that one can purchase on the high street” [19]) or could not be purchased in online or physical stores; (3) used a modified version in its mechanics or features of a COTS game that change a fundamental aspect of the game; (4) used custom-made games (ie, serious games); (5) did not specify the title of the game used; (6) did not specify the average age or age range of the participants.

### Search Terms and Selection of Papers for Inclusion

The search string was: [(“video game*”) OR (“computer game*”)] AND [(“stress”) OR (“anxiety”) OR (“relaxation”)] AND [(“study”) OR (“trial”) OR (“training”)]. Initially, two of the authors (FP and AP) checked the titles and abstracts of the identified articles to determine their eligibility. Subsequently, they independently reviewed the full texts of potentially eligible papers. Any disagreements between the two authors (FP and AP) were discussed until reaching a consensus. When papers provided insufficient data for inclusion in the analysis, the corresponding authors were contacted to provide additional data. No additional articles emerged via manual searching and reviewing the reference lists of relevant papers.

### Data Extraction

Two authors (FP and AP) independently extracted data on study characteristics and video game characteristics.

The study characteristics included the populations included in the study (participants, mean age or age range); study design (RCT, quasiexperimental, cross-sectional/correlational study); measures used for the assessment of outcomes (eg, self-report questionnaires, physiological data, cognitive task); study outcomes (ie, stress, anxiety, or both, and differences in the outcome measures related to playing COTS games). The populations, study design, measures of outcomes, and study outcomes were considered relevant variables according to the approach adopted in previous reviews [35,37,88,89] to facilitate easily classified and comparable studies in the literature. An indication of the mean age or age range identified studies performed with children (ie, under 12 years old), adolescents (12-18 years old), young adults (18-35 years old), middle-aged adults (36-55 years old), and older adults (over 55 years old). The division of these age ranges also followed previous studies [90-92].

The video game characteristics extracted included the game genre categorized as CVGs, action, adventure, racing, sports, role-playing game (RPG), strategy, simulation, exergames, and augmented reality (AR) (see Table 1); the platform for the game (console, mobile console, PC, smartphone/tablet, virtual reality); and time spent playing (duration and the total amount of sessions). Video game genre classification was considered because not all video games are equal from many aspects, and their effects strongly depend on specific characteristics of the game itself [93,94]. Since there is no standard accepted taxonomy of genre, although one of the most commonly adopted is the system proposed by Herz [95], the above categorization was proposed to be as similar as possible to the Entertainment Software Association (ESA) classification [14,96] (see Table 1). In addition to the ESA classification genres, AR games were added since they appear to be essential to the main research questions of this review [97]. Delivery platforms were considered since they represent important information about how computer games can be accessed. Since new technologies such as mobile devices and virtual reality have recently expanded how games are played, we further considered the delivery devices. Finally, in the studies that indicated play time, this information was included in the analysis, which can offer valuable insights about how and for how long to use COTS to effectively reduce stress and anxiety.

**Table 1:** Definitions of the main genres of video games adopted in the systematic review.

Video game genre	Definition	Examples
Action games	Require quick action and emphasize physical challenges, including hand-eye coordination and reaction time. This genre includes many subgenres such as fighting games, shooter games, and platform games	Super Mario Bros, Doom, Call of Duty, Mortal Kombat, Street Fighter
Adventure games	Characterized by complex plots and emphasize exploration and problem-solving. Typically, pure adventure games have situational problems for the player to solve, with very little or no action. If there is action, it generally includes isolated minigames	Zork, The Walking Dead, Until Dawn, Life is Strange, Heavy Rain, Beyond: Two Souls
Action-adventure games	A hybrid genre that combines core elements from both action and adventure game genres. These games require many of the same physical skills as action games, and offer a storyline, an inventory system, and other adventure games. This genre includes the subgenre of survival horror games, typically designed to scare the players	Tomb Raider, The Last of Us, Grand Theft Auto, Uncharted, Resident Evil, Left 4 Dead, Cyberpunk 2077
Casual video games	Short games with little or no plot that can be played in short sessions; they are quick and straightforward to learn	Bejeweled, Plants vs. Zombies, Tetris, FreeCell
Racing games	Racing competition with any vehicles (from real-world racing leagues to fantastical settings)	Gran Turismo, Need for Speed, Mario Kart
Sports games	Simulate the practice of sports, including team sports, combat sports, and extreme sports	FIFA 2020, NBA 2K20, Steep
Role-playing games	Players control an avatar and develop it over a certain period of time. This genre includes the subgenre of massively multiplayer online role-playing games, which are role-playing video games played online with large numbers of players	Final Fantasy, EverQuest, World of Warcraft, Dragon Quest, Diablo
Strategy games	Emphasize strategic thinking and resource management. This genre includes the multiplayer online battle arena. Each player controls a single character with unique abilities that improve throughout a game and contribute to the team’s overall strategy	Age of Empires, Civilization, Halo Wars, League of Legend, Dota II
Simulation games	Designed to closely simulate aspects of real life or fictional reality	The Sims, SimCity
Exergames	A combination of video gaming and physical exercise; these games require physical effort from the player to play the game	Just Dance, Ring Fit Adventure
Augmented reality games	Combine the use of mobile technology with physical exploration in the real world	Pokémon Go, Ingress

### Study Quality and Risk of Bias Assessment

The Mixed Methods Appraisal Tool (MMAT) [98] was used to assess the methodological quality of studies included in this systematic review. The MMAT has high reliability and efficiency as a quality assessment protocol and is capable of concomitantly appraising methodological quality across various types of empirical research [99]. Two authors (FP and AP) independently assessed study quality. Interrater reliability (Cohen κ=0.816) [100], calculated using the software package SPSS, demonstrated substantial agreement [101]. Disagreements on study quality were resolved by discussion between the two authors.

## Results

### Retrieved Articles

**Figure 1:**
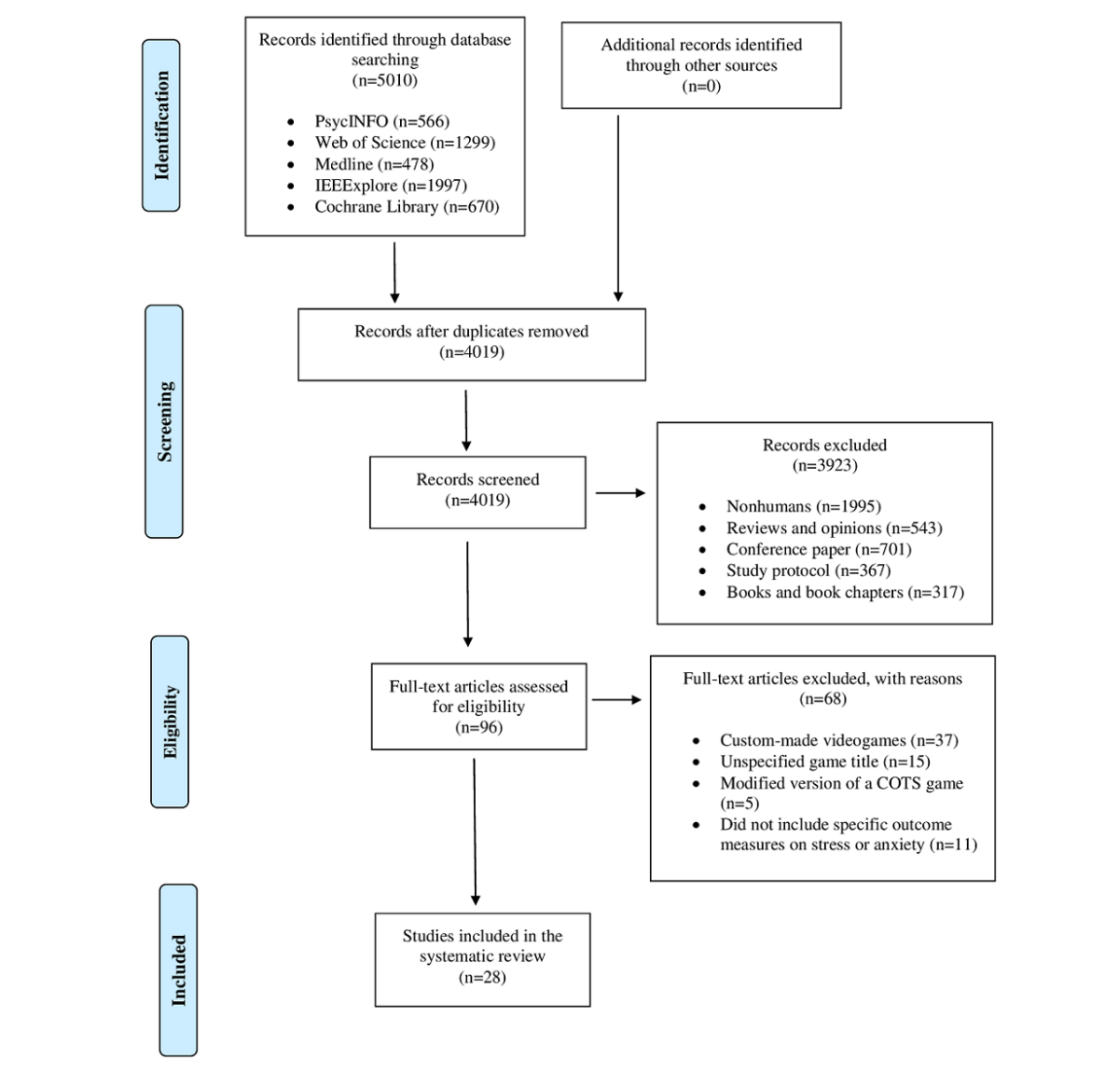
PRISMA flow chart of the systematic review. COTS: commercial off-the-shelf.

The search strategy retrieved 5010 records. After deduplication and language examination, 991 studies were excluded from the review process, and 3923 studies were excluded after the first screening and title and/or abstract analysis. Ninety-six full-text copies of the remaining articles were obtained and subjected to further evaluation. After reading the full text, 68 studies were excluded from this review for the following reasons: 11 studies did not focus on the use of COTS games for diminishing stress and/or anxiety, 26 used custom-made video games, 15 did not specify the game’s title, 5 adopted a modified version of a COTS game, and 11 did not include specific outcome measures on stress or anxiety. Finally, 28 studies remained for inclusion in the review (see Figure 1 and Multimedia Appendix 1).

### Quality Assessment Outcomes

An overall quality score was assigned to each study using the MMAT scoring system [98]. Studies could be awarded a score of 0, 25, 50, 75, or 100 (with 100 indicating the highest quality). The distribution of MMAT scores among the included studies varied substantially according to study design (Table 2).

Among the 28 studies, 5 (18%) scored 100, 12 (43%) scored 75, 5 (18%) scored 50, 5 (18%) scored 25, and 1 (4%) scored 0. See Multimedia Appendix 2 for details of quality assessment for each study.

Of the 17 studies employing a quantitative RCT methodology, 11 studies did not perform randomization appropriately (ie, inserted a simple statement such as “we randomly allocated”), 10 studies did not specify if outcome assessors were blinded to the intervention provided, and 5 studies did not check or specify if the groups were comparable at baseline.

**Table 2:** Study design and Mixed Methods Appraisal Tool (MMAT) quality score distribution.

MMAT score distribution		References	Studies, n (%)
**Quantitative randomized controlled trials (n=17)**			
	0	None	0 (0)
	25	[36,102-105]	5 (29)
	50	[106-108]	3 (18)
	75	[109-113]	5 (29)
	100	[85,86,114,115]	4 (24)
**Quantitative nonrandomized studies (n=8)**			
	0	[116]	1 (13)
	25	None	0 (0)
	50	[80]	1 (13)
	75	[117-122]	6 (75)
	100	None	0 (0)
**Quantitative descriptive studies (n=3)**			
	0	None	0 (0)
	25	None	0 (0)
	50	[123]	1 (33)
	75	[124]	1 (33)
	100	[82]	1 (33)

None of the 8 studies that used a quantitative nonrandomized methodology accounted for confounders in the design and analysis. Two of these studies did not clearly describe the target population and the sample (inclusion and exclusion criteria) [80,116]. One study did not adopt appropriate measurements and did not report complete outcome data [116].

Two of the three studies using a quantitative descriptive methodology did not report establishing an appropriate sampling strategy to address the research question [123,124] and one failed to ensure that the sample was representative of the population under study [123].

### Characteristics of Included Studies

#### Study Design

RCT was the design of choice for 17 of the included studies. Nine studies adopted a quasiexperimental design and 2 studies [82,124] used a cross-sectional/correlational research design (see Table 2).

#### Populations

The number of participants ranged from 27 [109] to 337 [85,111] in RCT studies and from 1 [123] to 40 [120] in quasiexperimental studies. The two cross-sectional/correlational studies included 3915 [82] and 133 participants [124], respectively. The majority of studies (n=19) recruited young adults, while four studies involved older adults [105,109,112,117], 3 studies involved children [103,110,123], and 2 studies focused on middle-aged adults [82,102]. No study that recruited adolescents emerged in our final article list (Table 3).

Fifteen studies involved healthy participants, in most cases recruited from university staff and students, whereas three studies recruited participants among full-time workers [82], soldiers [86], and older adults in federal programs for assistance [117]. The other studies included soldiers who had posttraumatic stress disorder [113]; older adults who lived in a nursing home [112] and with Parkinson disease [109]; adults with at least minimal symptoms of depression [114], clinical depression, and comorbid anxiety [102], systemic lupus erythematosus [122], obesity [108], physical disabilities [116], and hematologic malignancies [105]; women after emergency cesarean section [115]; children with molar incisor hypomineralization–affected teeth [103], chronic wounds on the lower limbs that require active dressing changes [110], and who sustained second- and third-degree burns to the shoulders, neck, chest, bilateral forearms, and left thigh [123] (Table 3).

**Table 3:** Characteristics of included studies (N=28).

Characteristics		References	Studies, n (%)
**Health conditions**			
	Healthy	[36,80,82,85,86,104,106,107,111,117-121,124]	15 (54)
	Dressing change pain	[110,123]	2 (7)
	Posttraumatic stress disorder	[113]	1 (4)
	Parkinson disease	[109]	1 (4)
	Minimal depression	[114]	1 (4)
	Clinical depression and anxiety	[102]	1 (4)
	Systemic lupus erythematosus	[122]	1 (4)
	Obesity	[108]	1 (4)
	Physical disabilities	[116]	1 (4)
	Hematologic malignancies	[105]	1 (4)
	Emergency cesarean section	[115]	1 (4)
	Routine dental treatment	[103]	1 (4)
	Institutionalized older people	[112]	1 (4)
**Study outcome**			
	Stress	[36,80,82,85,86,104,106-108,111,118,121,123,124]	14 (50)
	Anxiety	[102,103,105,109,110,112-114,117,119,120,122]	12 (43)
	Both stress and anxiety	[115,116]	2 (7)
**Age range**			
	Children (<12 years)	[103,110,123]	3 (11)
	Adolescents (12-18 years)	None	0 (0)
	Young adults (18-35 years)	[36,80,85,86,104,107,108,110,111,113-116,118-122,124]	19 (68)
	Middle-aged adults (36-55 years)	[82,102]	2 (7)
	Older adults (>55 years)	[105,109,112,117]	4 (14)
**Gender**			
	Both male and female	[36,85,102,104-107,109-112,114,116,117,119-121,124]	18 (64)
	Male only	[80,86,108,113]	4 (14)
	Female only	[115,120,122,123]	4 (14)
	Unspecified	[103,118]	2 (7)

#### Outcome Measures

All studies used self-reported quantitative measures of psychological constructs. Five studies used the State-Trait Anxiety Inventory [125], three studies [105,115,122] used the Hospital Anxiety and Depression Scale [126], and two studies [85,124] used the Perceived Stress Scale [127]. Ten studies included physiological measures, three adopted cognitive tasks [104,109,112], and four used other types of performance tasks [105,109,110,121].

#### Study Outcomes

##### Stress

Fourteen studies focused primarily on investigating COTS games for reducing stress (Table 3). Eight studies reported that COTS games were superior for reducing stress when compared with basic stress management training [86], guided relaxation or sitting quietly [104], a traditional exercise program at a moderate intensity [118], surfing the web [36], a passive video game distraction [123], a standard distraction procedure [110], and not playing games [85,111]. By contrast, a study including adult men with overweight/obesity reported increased stress levels after playing COTS games, which were higher than those recorded after watching nonviolent television [108]. Two studies compared the effects of different video games on reducing stress [106,121], showing that playing an action game elicited higher arousal and stress than playing a CVG [106,121]. Another study compared two versions (ie, cooperative vs competitive) of the same action-adventure game, showing a decrease in stress levels after both [107]. One study showed that although an action game increased stress, it also elicited happiness in players [80]. Finally, two studies investigated the relationship between stress and the use of some COTS games. Psychological stress was significantly reduced among Pokémon Go players than among nonplayers [82]. Moreover, a relationship emerged between stress levels and the use of the massively multiplayer online role-playing game (MMORPG) World of Warcraft. In particular, individuals with a low level of stress reported playing this game to enhance their offline lives. By contrast, highly stressed individuals reported that playing this game magnified rather than relieved their suffering [124].

##### Anxiety

Twelve studies reported outcomes for decreasing anxiety (Table 3). Nine studies reported improvement after playing a COTS game compared to an eye movement desensitization and reprocessing (EMDR) therapy alone [113], watching a film [117], surfing the web [114], anxiolytic medication [102], physiotherapy alone [105], passive video game distraction [123], and not playing a game [103,109,112]. Furthermore, in two studies that did not include a control group, exergames diminished anxiety in only one session [120] and in a more extended program including a total of 30 sessions [122]. Another study compared the efficacy of an exergame and a CVG played in virtual reality; anxiety reduction was more significant in the case of the exergame [119].

##### Combined Approach

Only two studies focused on both stress and anxiety in a combined manner (Table 3) [115,116]. In the first study, exergames were efficacious in reducing anxiety in a sample of individuals with physical disabilities; however, no differences emerged in stress or depression [116]. In the other study, self-reported acute stress symptoms and the frequency of intrusive traumatic memories after traumatic childbirth reduced after engaging in the brief cognitive intervention, including playing Tetris; however, no differences emerged regarding anxiety and depression [115].

### Video Game Characteristics

#### Genre

Twenty-four studies used only one video game, whereas the other four studies adopted two video games of different genres [106,108,119,121]. Twelve studies used exergames, whereas nine studies used CVGs. Four studies adopted an action game [80,106,108,121], which was a shooter game in three studies [80,108,121] and a fighting game in the other [106]. Three studies used action-adventure games [86,107,110], which was a survival horror game in one study [86]. The other studies used an MMORPG [124], sports game [108], racing game [103], and an AR game [82] (see Table 4).

**Table 4:** Video game characteristics (N=28).

Characteristics			References		Studies, n (%)
**Genre**					
	Casual video games	[36,102,104,106,113-115,119,121]		9 (28)	
	Exergames	[85,105,109,111,112,116-120,122,123]		12 (38)	
	Action games	[80,106,108,121]		4 (13)	
	Role-playing games	[124]		1 (3)	
	Action-adventure games	[86,107,110]		3 (9.4)	
	Sports games	[108]		1 (3)	
	Racing games	[103]		1 (3)	
	Augmented reality games	[82]		1 (3)	
**Platform**					
	PC	[36,80,86,102,104,106,114,121,124]		9 (32)	
	Console	[85,105,107-109,111,112,116-118,120,122,123]		13 (46)	
	Smartphone	[82]		1 (4)	
	Mobile console	[113,115]		2 (7)	
	Virtual reality	[103,110,119]		3 (11)	
**Total time of play (minutes)**					
	<10	[103,104,119,121]		4 (16)	
	11-60	[36,80,85,106-111,115-117,120,123]		14 (48)	
	61-180	[86,105,118]		3 (12)	
	>180	[102,112-114,122]		5 (16)	
	Unspecified	[82,124]		2 (8)	

#### Platform

Games delivered via console were the most popular, with 13 studies using this game platform (Table 4). In particular, 7 studies used Nintendo Wii Fit, 5 studies used Microsoft Xbox 360 with Xbox Kinect, 1 study used tXbox One [107], and 1 study used Sony PlayStation 3 [108]. Nine studies used a PC, three studies

used a virtual reality viewer [103,110,119], one study used a smartphone [82], and two studies used a portable console (ie, Nintendo DS and Nintendo DS XL) [113,115].

#### Time of Play

In the 26 studies that measured the effect of time playing COTS games on stress and anxiety levels, there was a heterogeneous result. The mean number of sessions was 6.6, ranging from 1 session (eg, [107,116,121]) to 30 sessions [122].

The actual time spent playing video games differed among studies, ranging from about 2 minutes [103] to up to 15 hours [112,122]. Only two studies (ie, the two cross-sectional studies) did not indicate the exact playing time (Table 4).

## Discussion

### Principal Findings

This systematic review examined studies performed to investigate the efficacy of COTS video games for diminishing stress and anxiety. After applying the inclusion criteria, 28 papers were included for analysis. Most studies were published after 2014, with many studies (almost 40%) published after 2018. Interest in this field was crucially fueled by publication of the first study on this topic in 2009 [36]. Seventeen studies (61%) met the MMAT quality assessment score of 75% or above. This suggests that much of the research in this area is of high quality; nevertheless, the quality scores varied substantially according to the study design.

With respect to the population of focus, the majority of studies involved young adults (ie, 18-35 years). This finding also emerged in a previous systematic review on the use of video games, including COTS and custom-made games, to train cognitive skills [20]. A possible explanation of this tendency could be that many studies have enlisted college students as participants for recruitment simplicity.

Three studies involved middle-aged adults (ie, 36-55 years old) [82,102,113]. Based on emerging results, the use of COTS games can offer essential support for people of this age group, who, besides representing the most significant percentage of video game players [14], are particularly susceptible to high stress and anxiety [128,129].

Three studies recruited older adults (ie, up to 55 years old). The results of these studies suggest that the use of COTS games can be helpful for the elderly population not only to improve cognition [130-132] or to enhance physical activity [133-135] but also for relaxation [105,109,117]. This fact appears relevant since, if older adults generally do have lower stress and anxiety and better emotional regulation than younger adults [136], given the COVID-19 pandemic, this age group is currently experiencing significant adverse psychological consequences [134,135,137]. The COVID-19 pandemic has exacerbated stressors for older adults because of the risk of becoming seriously ill and the need for social isolation to mitigate this risk.

With respect to younger age groups, two studies recruited children (ie, under 12 years old) [110,123]. Playing COTS games, mainly through consoles (ie, Nintendo Wii), helped to diminish anxiety and alleviated pain in even very young children during painful or invasive medical procedures such as burn dressing changes and dental treatment. This fact appears to be important because there is a need for therapeutic alternatives within this age range with relatively limited medication options [138].

Finally, no study emerged specifically focusing on adolescents (ie, 12-18 years old). A possible explanation could be related to the intense debate in the scientific community and the general public about the effect of video games, especially those characterized by high levels of violence, on young people’s mental health [139,140].

Concerning the health characteristics of the participants included in the studies, interestingly, COTS video games reduced stress and anxiety not only in healthy individuals (eg, [106,118,141]) but also in patients suffering from different mental disorders such as posttraumatic stress disorder [113]; Parkinson disease [109]; depression [102,114]; comorbid anxiety [102]; as well as physical problems such as physical disabilities [116], systemic lupus erythematosus [122], hematologic malignancies [105], or severe burns [123].

Regarding the experimental design, this systematic review showed that most studies (almost 60%) used an RCT design. Future studies should continue using this type of experimental design, representing the most reliable empirical design to prove a treatment’s effectiveness, thereby minimizing the impact of confounding variables [142].

The outcome measures adopted in the studies included in this systematic review predictably primarily constituted self-administered psychological questionnaires, which were used in all studies. Nonetheless, numerous studies also included physiological measures (eg, heart rate variability, blood pressure, concentration of salivary cortisol), cognitive tests, and performance tasks, which seem to be more reliable in assessing change over time. Therefore, openness to different methods of assessment is desirable from the perspective of empirical evidence. Moreover, since many different tools are used, especially self-report questionnaires, in the future, it will be essential to define a set of standard measures for the evaluation of stress and anxiety that are specific to the different age ranges of participants.

With respect to outcomes, 14 studies included in this systematic review primarily focused on investigating COTS games for reducing stress, 12 focused on anxiety, and 2 assessed both of these conditions [115,116]. Empirical evidence emerged concerning the efficacy of COTS video games in reducing both stress and anxiety.

COTS games appear to be superior for reducing stress when compared with both control procedures (eg, sitting quietly or surfing the web) [85,110,111,123] and traditional techniques such as stress management training [86], guided relaxation [104], or a standard distraction procedure [110]. An action-adventure game (ie, Lego: Marvel Superheroes) decreased stress both in the cooperative and competitive versions [107]. In the two studies investigating the relationship between stress and the use of some COTS games, psychological stress was significantly more reduced among Pokémon Go players than among nonplayers [82]. In addition, a relationship emerged between stress levels and use of the MMORPG game World of Warcraft [124].

However, other studies reported no decreases in the levels of stress of the players. A more significant increase in stress levels emerged after playing FIFA 2013 and Call of Duty compared with the levels recorded after watching nonviolent television [108]. Playing an action game, specifically a shooter game (ie, Counter-Strike), elicited an arousal stress response but also increased happiness in players [80]. Furthermore, playing action games (ie, Mortal Kombat: Komplete Edition, Light Heroes) elicited higher levels of stress than CVGs (ie, Tetris Ultimate, Clusterz) [106,121].

Concerning anxiety, studies that emerged from this systematic review reported better improvement after playing a COTS game compared with not playing the game [103,109,112], surfing the web [114], watching a film [117], a passive video game distraction [123], EMDR therapy [113], anxiolytic medication [102], or physiotherapy alone [105]. Furthermore, two studies found a significant decrease in anxiety after a single exergame session [120] and after an exercise program with the same video game genre [122].

Regarding studies examining both stress and anxiety, in the first, anxiety, but not stress or depression, decreased after an intervention using exergames in a sample of individuals with physical disabilities [116]. In the second, playing a CVG (ie, Tetris) within a brief cognitive intervention reduced the frequency of intrusive traumatic memories after emergency cesarean section, but did not affect anxiety or depression [115].

With respect to video game characteristics, considering game genre distribution, exergames were the most frequently used, closely followed by CVGs. This result is partly surprising, as previous literature focused almost exclusively on CVGs to reduce stress and anxiety. This genre of games has proven to be able to diminish state anxiety [35,102,114] as well stress of the players [36], even to a greater extent than medical treatment [114]. In particular, playing a CVG under a prescribed condition added to an individual’s medication regimen significantly reduced state anxiety symptom severity and had a medium effect on trait anxiety compared with the medication intervention alone [114].

However, based on the findings that emerged in this systematic review, CVGs are not the only promising genre for decreasing players’ stress and anxiety. As noted above, many studies included in this review used different genres, especially exergames. Owing to their high level of interactivity and high-quality entertainment [143-145], exergames represent one of the most appealing video game genres for inducing positive emotions and decreasing stress and anxiety. In addition to exergames and CVGs, this systematic review showed that other genres of COTS video games could also be helpful for the reduction of stress and anxiety, including action games [80,106,108,121], particularly shooter games [80,106,121] and a fighting game [106]; action-adventure games [86,107,110], including survival horror games [86]; RPGs, in particular MMORPGs [124]; sports games [108]; racing games [103]; and AR games [82].

Concerning action games, and in particular shooter games, a study performed using Counter-Strike reported a high physiological arousal response, accompanied by the perception of a positive emotional state and decreased negative emotions [80]. Based on this result, it appears possible that shooter games activate an intense arousal response in the player while improving their emotional state, likely because they require high cognitive resources [146,147]. However, this hypothesis requires further investigation.

Two other studies included in this review showed an increase in stress levels and a physiological arousal response after playing a shooter game (ie, Call of Duty), which were higher than those measured after watching nonviolent television [108] or playing a CVG (ie, Clusterz) [121].

This systematic review also offers evidence about the efficacy of action-adventure games for reducing stress, not only in young adults but also in children [86,107,110]. This fact is interesting because this genre of games includes titles suitable for children, such as those used in the two studies that emerged from the review (ie, Ice Age 2: Meltdown and Lego: Marvel Superheroes). A subgenre of action-adventure video games that reduced stress and anxiety in young adults was survival horror.

One study performed on young adult male soldiers reported that playing a horror game (ie, Left 4 Dead) combined with biofeedback techniques reduced stress to a greater extent than training as usual [86]. Therefore, together with exergames and CVGs, horror games could represent another game type for effectively managing stress and anxiety.

One study in this review provides preliminary evidence that racing games could help decrease the players’ stress and anxiety, especially in children [103]. This video game genre shares many characteristics with CVGs, such as ease of learning and short duration. For this reason, it would be interesting to further explore the use of racing games for the reduction of stress and anxiety in other age groups such as adolescents and adults who could also obtain a benefit.

Based on the results of this review, another interesting genre to help reduce stress and anxiety is AR games [82], which combines smart mobile technology with physical exploration in the real world. In particular, the players of Pokémon Go, one of the most famous titles in this category released in 2016, reported a lower level of stress than nonplayers. These findings seem very intriguing since, unlike most video games, AR games such as Pokémon Go have unique features that encourage social interaction and physical movement [148-150]. They may have a possible therapeutic role in helping stressed or anxious people deal with their everyday experiences.

Regarding the platform, 11 of the included studies delivered games via a console, especially Nintendo Wii Fit and Microsoft Xbox 360 with Xbox Kinect. Curiously, one of the most famous and popular consoles (ie, Sony PlayStation) was used in only one study in the PlayStation 3 version [108]. No study has used recent versions of this console, namely PlayStation 4 and PlayStation 5, released in 2013 and 2020. Nine studies included in the review used a PC as the platform and virtual reality systems were used in three studies [103,110,119]. Only one study adopted smartphones [82] or mobile consoles [113].

Finally, the effect of time of play was heterogeneous, both in terms of the number of sessions and the specific time spent playing video games. In particular, the number of sessions ranged from a minimum of 1 (eg, [36,110,117] to a maximum of 30 over 10 weeks [122], and the total playing time varied from a few minutes [102] to over 15 hours [122]. The fact that even single and short sessions (ie, 1 or 5 minutes) of play were effective in reducing stress and anxiety appears particularly interesting.

### Potential Risks in Using COTS Video Games for Relaxation

In addition to offering data in favor of the effectiveness of COTS video games in reducing stress and anxiety, the results of this review also raise some critical reflections on the possible risks of using these games for this aim.

First, COTS games appear to be not always useful for relaxation. Some studies included in this review reported an increase in stress after playing a sports game (ie, FIFA 2013) [108], as well as a more intense stress and arousal response after an action game, in particular a fighting game (ie, Mortal Kombat: Komplete Edition), than a CVG (ie, Tetris Ultimate) [106]. In another study using an MMORPG (ie, World of Warcraft), highly stressed individuals reported that playing this game magnified rather than relieved their suffering [124].

Second, some gaming platforms are not suitable for all ages. In particular, concerning the use of video games played in virtual reality, it is important to emphasize the possible risks for children under 12 years old [151]. As indicated by all of the manufacturers of head-mounted displays, including Oculus VR, use in individuals under 12 years old is not recommended [151]. This decision connects to the fact that children are more vulnerable to virtual reality, as they are highly susceptible and can more easily confuse what is real and what is not real; thus, children may be less able or unable to distinguish between the real world and the virtual world [152,153]. To date, only one study focused on the safety of current virtual reality devices for children with respect to possible negative consequences on children’s eyes [154].

### Limitations

This review does not claim to be comprehensive but rather summarizes the research on COTS video games for reducing stress and anxiety based on specific keywords used in the search string, the databases searched, and the time period under analysis. Moreover, this review analyzed video games using a specific categorization of their genres, although the best approach to classify video games is an ongoing discussion. Therefore, it is essential to emphasize the specificity of the classification used, which resembles the video games’ ESA classification as much as possible [14,96]. In addition, the included studies presented high heterogeneity for stress and anxiety levels and the recruited sample regarding age and health conditions. There was also considerable heterogeneity found in the COTS game genres, platforms, times of play, and methods used to assess stress and anxiety levels among the included studies. Therefore, the results from this systematic review require careful interpretation.

### Future Directions

This systematic review provides several directions for future studies in this research field. First, given that COTS video games are used not only by young adults but also by people of all age groups [14], it seems necessary to further explore the use of video games to reduce stress and anxiety in diverse populations, especially in younger and older individuals. Furthermore, future studies should investigate the effectiveness of COTS video games in adolescents, a population that was not involved in any of the studies included in this review. This fact seems essential since the use of video games can favor adolescent adherence to psychological support programs more than traditional psychotherapy [155], and because young individuals often suffer from high levels of stress and anxiety, especially in this particular historical moment linked to the COVID-19 pandemic [155-157].

Second, since few studies performed to date used COTS games of a genre other than exergames or CVGs, future studies are needed to explore the efficacy of other genres in reducing stress and anxiety. In particular, based on the results that emerged from the review, the action, action-adventure, RPG, sports, racing, and AR games appear to be particularly interesting in this regard.

Third, these studies often adopted gaming platforms that are dated or not very accessible to the public. Future studies should also adopt more popular and widely used gaming devices (eg, PlayStation 4 or the newest PlayStation 5, Oculus Quest, or Oculus Quest 2). It also seems essential to investigate improved mobile gaming in the future, which could offer unique advantages over traditional tools such as a PC or console because of its potential ubiquity and real-time use.

Fourth, the most effective number of sessions and playing time required to achieve relaxation remain unclear. Future studies should address such aspects in detail, for instance by comparing shorter and longer times of play to identify the optimal playing time for reducing stress and anxiety. It also seems to be essential to verify how long the benefits of COTS games on anxiety and stress can last through follow-up studies.

Fifth, the quality assessment performed using the MMAT suggests that even if much of the research in this area is of high quality, methodological concerns are a significant issue for many studies. Researchers should follow reporting guidelines to ensure the completeness of the dissemination of research findings.

Finally, future studies are needed to explore the relationship between the effectiveness of COTS games for reducing stress and anxiety, and individual preferences concerning the genre and the gaming platform. In fact, such characteristics can have a meaningful impact on the efficacy of specific video games for diminishing stress and anxiety [58,158,159]. Furthermore, in the future, it will be necessary to investigate how other individual characteristics may influence the efficacy of COTS games in reducing stress and anxiety, including personality and cognitive ability.

### Implications for Clinical Practice

The findings of this review have some practical implications for health care practitioners. The COTS games that have more experimental evidence for their effectiveness in reducing stress and anxiety are the exergames and CVGs. Even short sessions of playing (eg, 1 or 5 minutes) can be helpful for relaxation. It is possible to use COTS video games for reducing stress and anxiety not only in healthy people but also in individuals with several mental and physical health problems. Finally, when selecting the gaming platform, it is essential to consider the player’s age (ie, avoiding virtual reality for children under 12 years of age).

### Conclusions

To summarize, this systematic review provides evidence of the benefits of COTS video games for reducing stress in children, young adults, and older adults. Efficacy has been demonstrated not only for exergames and CVGs but also for other genres of video games including action games, action-adventure games, and AR games. Various gaming platforms (ie, consoles, PCs, smartphones, portable consoles) showed positive results, including the most innovative platforms represented by virtual reality systems. Given their low cost and popularity among millions of players worldwide, COTS games may be an important tool in reducing stress and anxiety for many individuals, diminishing the existing psychological support barriers.

## Appendix

Multimedia Appendix 1 Description of the studies included in this review.

Multimedia Appendix 2 Quality assessment scores using the Mixed Methods Appraisal Tool (MMAT).

## Abbreviations

AR: augmented reality
COTS: commercial off-the-shelf
CVG: casual video games
EMDR: eye movement desensitization and reprocessing
ESA: Entertainment Software Association
MMAT: Mixed Methods Appraisal Tool
MMORPG: massively multiplayer online role-playing games
PRISMA: Preferred Reporting Items for Systematic Reviews and Meta-Analysis
RCT: randomized controlled trial
RPG: role-playing game
