# Supplementary material for: Commercial Off-The-Shelf Video Games for Reducing Stress and Anxiety: Systematic Review
Source: JMIR Ment Health. 2021 Aug 16;8(8):e28150. doi: 10.2196/28150 (PMC8406113; doi:10.2196/28150)
Supplement: Multimedia Appendix 1 [file mental_v8i8e28150_app1.docx]

| **Study** | **Aim** | **Sample** | **Age** | **Study Design** | **Video Game** | **Genre** | **Platform** | **Time spent playing** | **Outcome measures** | **Main outcomes** |
| --- | --- | --- | --- | --- | --- | --- | --- | --- | --- | --- |
| Alves et al [109] | To investigate changes in motor and cognitive skills, anxiety levels, and quality of life perception among patients with Parkinson’s Disease (PD) following  training with different commercial gaming devices (Nintendo Wii and Xbox Kinect). | 27 participants recruited from patients with PD | 61 ± 10.7 years old | Randomized controlled research design  Three conditions: Two Experimental Groups (Xbox Kinect and Nintendo Wii), and Control Group (no video game) | *Wii Fit Plus*  (Rhythm Parade, Obstacle Course,  Tightrope Walk,  Basic Step) | Exergames | Console (Nintendo Wii) | Three or five times per game for a total of about 15 minutes in each of the ten-session | Performance task:  gait performance test  Cognitive task:  Digit Span Forward and Backward, Verbal Fluency Test (VFT)  Self-report questionnaires:  Beck Anxiety Inventory (BAI), World Health Organization Quality of Life for Older Persons (WHOQOLOLD) | Only participants engaged with the  Nintendo Wii decreased anxiety levels, significantly improved their performance on single and dual-task gait tests, and improved memory, attention, and reversibility. The control group showed no changes on any measures. |
|  |  |  |  |  | *Kinect Sports* (Hurdles)  *Kinect Adventures* (River Rush, Reflex Ridge),  *Your Shape Fitness Evolved* (Light Race) |  | Console  (Microsoft Xbox 360 with Xbox Kinect) |  |  |  |
| Bouchard et al [86] | To assess the efficacy of using visual and auditory biofeedback while playing a videogame to practice stress management training (SMT) (i.e., tactical breathing). | 41 participants recruited from soldiers who had previously received basic stress management training and first aid training in combat | 24.9 ± 3.9 years old | Randomized controlled research design  Two conditions: Experimental Group (biofeedback-assisted SMT while immersed in a horror game), and Control Group (no additional SMT) | *Left 4 Dead*  combined with biofeedback | Action video game  (survival horror video games) | PC | Three 30-minute sessions, one per day for three days | Physiological: concentration of salivary cortisol, heart rate  Self-report questionnaires: Perceived self-efficacy, medical  instructors’ ‘‘blind’’ evaluation of participant’s performance during  the simulation | Results showed a significant difference in cortisol response, documenting that practicing stress management training while playing diminished stress more than training as usual. |
| Butler et al [113] | To investigate Tetris as a preventive intervention to reduce intrusive memories of a traumatic event in patients with existing posttraumatic stress (PTSD) and explore how playing this video game may affect brain structure. | 40 participants with combat-related PTSD from the German Federal Armed Forces before they started therapy. All participants were male and had been deployed overseas to areas of conflict | 33.3 ± 6.3 years old | Randomized controlled research design  Two conditions: Experimental Group (Tetris and Eye Movement Desensitization and Reprocessing therapy), and Control Group (EMDR therapy only) | *Tetris* | Casual video game (puzzle video games) | Mobile console (Nintendo DS XL) | 60 minutes per day for two sessions per week, for six weeks | Physiological:  MRI (neuroimaging) scanner system  Self-report questionnaires:  Posttraumatic Diagnostic Scale (PDS), Beck Depression Inventory-II (BDI-II), State-Trait Anxiety Inventory Form Y-2 (STAI-Y2) | Following therapy, hippocampal volume increased in the Tetris group but not the control group. Hippocampal increases correlated with reductions in symptoms of PTSD, depression, and anxiety between completion of therapy and follow-up in the Tetris group, but not the control group. |
| De Morais [117] | To investigate the effect of exercise through video games on mood and physical activity in older adults. | 29 older adults recruited in federal programs of assistance for older adults. | 66.4 ± 0.8 years old | Quasi experimental, pretest-posttest design  Two conditions: Exercise Condition (exercising with the Xbox Kinect), and Control Condition (watching a film) | *Your Shape Fitness Evolved* | Exergame | Console  (Microsoft Xbox 360 with Xbox Kinect) | 60 minutes in total in one session | Self-report questionnaires:  Profile of Mood States (POMS), Physical Activity Enjoyment Scale (PACES), Borg Perceived Exertion Scale (BPES) | Regarding the mood states, no differences emerged comparing exercise and control conditions, but significant within-subjects reductions emerged in the following POMS subscales: tension-anxiety, depression. Moreover, higher enjoyment during the exercise in the Xbox Kinect was associated with greater improvement in mood. |
| Fish et al [114] | To test whether a prescribed casual videogame play regimen could reduce individuals' anxiety symptom severity in a depressed population. | 59 participants recruited from adults experiencing at least minimal symptoms of depression | 30  years old | Randomized controlled research design  Two conditions: Experimental Group (playing casual video games), and a Control Group (engaging in a web-based review on depression) | *Bejeweled 2, Bookwarm Adventures,*  *Peggle* | Casual video games | PC | A minimum of 30 minutes per session, three times each week, for one month | Self-report questionnaires:  State-Trait Anxiety Inventory Form Y-1 (STAI-Y1), STAI-Y2 | A prescribed regimen of casual video games play significantly reduced state and trait anxiety symptom severity as measured by the STAI. |
| Fish et al [102] | To examine prescribed casual video gameplay (CVG) added to a Selective Serotonin Reuptake Inhibitor (SSRI) prescription to reduce anxiety symptoms compared to a two medications treatment group. | 54 participants with clinical depression and co-morbid anxiety were prescribed SSRI and received psychiatric services for the reduction of their symptoms | 41.5  years old | Randomized controlled research design  Two conditions: Alternative Intervention Group (prescribed CVG), and Traditional Intervention Group (augmentative medication, such as anxiolytic medication) | *Plant vs. Zombies* | Casual video game | PC | 30-45 minutes, four times per week, for a total of 16 sessions in 4 weeks | Self-report questionnaires:  STAI-Y1, STAI-Y2 | Results showed a significant decrease in state anxiety scores after the one-month prescription and a medium to large effect for the alternative group. |
| Horsch et al [115] | To evaluate whether the number of intrusive traumatic memories mothers experience after emergency cesarean section (ECS) could be reduced by a brief cognitive task procedure, including playing a casual video game. | 56 participants recruited from women during the first 6 h following ECS and while still in the hospital environment. | 33.3 ± 4.2 years old | Randomized controlled research design  Two conditions: Experimental Group (Tetris condition), and Control Group (no-task control condition) | *Tetris* | Causal game | Mobile console  (Nintendo DS) | At least 15 minutes in one session | Self-report questionnaires: Hospital Anxiety and  Depression Scale (HADS), Acute Stress Disorder Scale (ASDS) | The mean number of self-reported acute stress disorder symptoms and the frequency of intrusive traumatic memories after traumatic childbirth diminished after engaging in the brief cognitive intervention, including playing Tetris. No differences emerged regarding anxiety and depression. |
| Hua et al [110] | To investigate the effect of virtual reality distraction on alleviating pain during dressing changes in children with chronic wounds on their lower limbs. | 65 participants recruited from a pediatric center aged 4-16 years and with chronic wounds on the lower limbs that require active dressing changes | 8.7  years old | Randomized controlled research design  Two conditions: Experimental Group (virtual reality game distraction), and Control Group (standard distraction) | *Ice Age 2: The Meltdown* | Action-adventure game | Virtual reality (eMagin Z800 3DVISOR) | About 30 minutes in one session | Performance task:  time length of dressing  Physiological:  pulse rate and oxygen saturation  Self-report questionnaires:  Wong-Baker Faces Picture Scale, Visual  Analogue Scale (VAS), Pain Behavior Scale (PBS) | Virtual reality distraction significantly relieved pain and anxiety scores during dressing changes and reduced the time length for dressing changes compared to standard distraction methods. |
| Huang et al [85] | To examine how playing exergames impacts university students and staff's mood states and whether such an impact depends on gender and players' previous exercise time. | 337 participants who were volunteers recruited from university students and staff at a university in northern Taiwan. | At least 20 years old | Randomized controlled research design  Two conditions: Experimental Group (exergames), and Control Group (no exergames) | *Your Shape Fitness Evolved* | Exergames | Console  (Microsoft Xbox 360 with Xbox Kinect) | 30 minutes, once a week for two weeks | Self-report questionnaires:  a shortened version of the Profile of Mood States (POMS), adapted version of the  Subjective Happiness Scale (SHS), Perceived Stress Scale (PSS) | Playing exergames reduces perceived stress and enhanced vigor and happiness. This effect of playing exergames was not moderated by gender, age, occupation (student or staff), or previous exercise time. |
| Huang et al [111] | To test whether playing exergames makes players perceive more energetic and relaxed and whether enthusiasm about doing exercise moderate such perceptions. | 337 participants recruited from university students and staff | At least 20 years old | Randomized controlled research design  Two conditions: Experimental Group (exergames), and Control Group (no exergames) | *Your Shape: Fitness Evolved* | Exergames | Console (Microsoft Xbox 360 with Xbox Kinect) | 30 minutes, once a week for two weeks | Self-report questionnaires: Profile Mood States (PMS), Subjective Happiness Scale (SHS), perceived energy (the perception of sufficient physical and mental resources) | Playing exergames induces positive changes in happiness, perceived energy, and relaxation. Such changes are significant for enthusiastic participants about doing exercise but not for those unenthusiastic about exercising. |
| Jahouh et al [112] | To determine the impact and effectiveness of using exergames on improving mood, anxiety, and cognitive impairment levels in institutionalized older people. | 80 participants recruited from older adults who lived in a nursing home | 84 ± 8.6 years old | Randomized controlled research design  Two conditions: Experimental Group (exergames), and Control Group (no exergames) | *Wii Fit Plus*  (Basic Step, Nodding) | Exergames | Console (Nintendo Wii) | 20 sessions, over eight consecutive weeks for about 45 minutes each | Cognitive assessment:  Lobo’s Mini-Cognitive Examination (MCE)  and Global Deterioration Scale (FAST-GDS)  Self-report questionnaires: Dementia  Apathy Interview and Rating (DAIR), Yesavage scale for Geriatric Depression (EGD-15), Goldberg  Anxiety and Depression Scale (EADG) | Playing exergames reduced anxiety, depression, and apathy levels and increased cognitive status in the elderly. |
| Naugle  [118] | To compare the emotional and cardiovascular effects of Wii games with those of traditional exercise in college-aged adults with different exercise backgrounds. | 22 participants recruited from the university campus, who were college-aged recreationally active volunteers | 20.5 ± 1.1 years old | Quasi experimental, pretest-posttest design  Two conditions: Experimental Condition (Nintendo Wii games), Control Condition (traditional exercises) | *Wii Sport*  (Boxing, Tennis, Step, Cycling) | Exergames | Console  (Nintendo Wii Fit) | Three exercise sessions, with 2 exercise activities performed during each session for 20 minutes each. | Physiological: salivary cortisol, heart rate  Self-report questionnaires:  Godin Leisure-Time Exercise Questionnaire (GLTEQ), Positive and Negative Affect Schedule (PANAS) | The Wii games produced a reduced cardiovascular response compared to the traditional forms of physical activity. Wii boxing and Wii tennis elicited the highest enjoyment levels and increased positive emotions after gameplay. |
| Özükoç [103] | to evaluate the effects of virtual reality video games on anxiety levels during dental treatments in children with molar incisor hypomineralization (MIH) affected teeth. | 28 children between the ages of 8–12 years selected among children who came to our clinic for routine dental treatments. | Between the ages of 10 and 12 | Randomized controlled research design  Two conditions: Virtual Reality Group 1, and Virtual Reality Group 2 (the order of VR exposure was reversed) | *InCell* | Racing video game | Virtual reality  (Preo VR Box cardboard) | About 2 minutes (during the venipuncture procedure) | Self-report questionnaires:  Children’s Perioperative Multidimensional Anxiety Scale (CPMAS) | Children undergoing treatment while playing virtual reality games experienced less dental anxiety. They were less worried and fearful during their appointments, exhibited less nervousness, and did not worry about future discomfort. |
| Pallavicini & Pepe [119] | To explore the ability of commercial virtual reality games to induce positive emotions and diminish negative emotions and state anxiety of the players, investigating the effects of the level of body involvement requested by the game (i.e., high vs. low). | 36 participants recruited from universities students and personnel via flyers distributed to campuses and word of mouth | 25.6 ± 4.2 years old | Quasi experimental, pretest-posttest design  Two conditions: High Body-Involvement Video Game (Audioshield), and Low Body-Involvement Video Game (Fruit Ninja VR) | *Audioshield* | Exergames | Virtual reality  (HTC Vive system) | About 5 minutes game in one session | Self-report questionnaires: VAS happiness, surprise, fear, sadness and anxiety, STAI-Y1 | Virtual reality video games appear to be practical tools to elicit positive emotions and decrease negative emotions and state anxiety. Besides, the virtual video game's level of body involvement had an important effect in determining the game's ability to improve positive emotions and decrease negative emotions and state anxiety. |
|  |  |  |  |  | *Fruit Ninja VR* | Casual video game |  |  |  |  |
| Porter & Goolkasian [106] | To investigate whether the emotional and cardiovascular changes associated with gameplay would be influenced by video game content. | 148 participants recruited from psychology students | 19.9 ± 3.2 years old | Randomized controlled research design  Two conditions: Fighting Game Group, and Puzzle Game Group | *Mortal Kombat: Komplete Edition* | Action video game (fighting game) | PC | 15 minutes in one session | Physiological:  blood pressure and heart rate variability  Self-report questionnaires: Biopsychosocial Model of Challenge and Threat, primary appraisal scales, positive and negative emotion ratings | Increased blood pressure and decreased heart rate variability were associated with fighting game players compared to the puzzle game players, indicating a cardiovascular stress response. However, fighting game players also reported higher positive emotion ratings. |
|  |  |  |  |  | *Tetris Ultimate* | Casual video game (puzzle game) |  |  |  |  |
| Roy & Ferguson [107] | To investigate the effect of playing a video game (either competitively or cooperatively) to recover from acute stress. | 100 participants who were undergraduate students from a small, liberal arts university | 18-25 years old | Randomized controlled research design  Two conditions: Cooperative Play Group, and Competitive Play Group | *Lego: Marvel Superheroes* | Action-adventure game | Console  (Xbox One) | 15 minutes in one session | Physiological:  blood pressure and heart rate  Self-report questionnaires: Psychological Stress Measures Questionnaire (PSM) | Stress levels declined over time at equal levels during both competitive and cooperative gameplay. |
| Rupp et al [104] | To investigate the effects of a passive break, relaxation activity, and casual video game on affect, stress, engagement, and cognitive performance. | 66 participants recruited from undergraduate students with normal or corrected-to-normal acuity and color vision | 20.3 ± 2.7 years old | Randomized controlled research design  Three conditions: Video Game Group, Guided Relaxation Group, and Control Group (sitting quietly) | *Sushi Cat 2* | Casual video game | PC | 5.5 minutes in one session | Cognitive assessment: Backward Digit-Span (BDS)  Self-report questionnaires: Positive and Negative Affect Schedule (PANAS), Dundee Stress State Questionnaire (DSSQ) | Participants who played the casual video game exhibited greater engagement and affective restoration in response to stress than the guided relaxation condition. |
| Russoniello et al [36] | To test the effects of casual video games on mood and stress by comparing people playing casual games with control subjects under similar conditions. | 134 participants recruited from fliers placed in and around the campus community | 24  years old | Randomized controlled research design  Two conditions: Experimental Group (playing casual video games), and Control Group (surfing the internet to look for articles related to health) | *Bejeweled 2, Bookwarm Adventures, Peggle* | Casual video games | PC | 20 minutes in one session | Physiological:  heart rate variability, electroencephalography  Self-report questionnaires: Profile of Mood States (POMS) | Electroencephalography changes during gameplay were consistent with increased mood and corroborated findings on psychological reports. Moreover, heart rate variability changes were consistent with autonomic nervous system relaxation or decreased physical stress. |
| Shin et al [80] | To investigate the efficacy of ‘‘Counter-Strike’’ to elicit positive emotions assessing the alpha asymmetry index (AI). | 12 participants who were right-handed male university students with normal or corrected-to-normal vision. | 25.1 ± 3.0 years old | Quasi-experimental, pretest-posttest design  Five conditions: Experimental Condition (playing CS game), Control Condition (sitting on a chair staring at a blank wall), Condition 1 (moving fingers on the computer keyboard), Condition 2 (listening to the sound of the CS game), and Condition 3 (watching the CS game without playing) | *Counter-Strike* | Action video game (shooter video game) | PC | Ten rounds of Counter-Strike one session for a total of about 20 minutes | Physiological: electroencephalography  Self-report questionnaires: subjective ratings of emotional states of happiness, sadness, anger, fear, arousal level | Participants’ ratings showed significantly greater happiness during the game and postgame compared with the baseline condition. In contrast, both the anger and arousal scores were significantly higher during the game than pre- or postgame. The ratings for sadness and fear were not significantly different between conditions. |
| Schumacher et al [105] | To investigate the influence of exergaming or classical physiotherapy on physical fitness and psychological well-being. | 49 participants recruited from patients receiving hematopoietic stem cell transplantation (HSCT) for hematologic malignancies | 56  years old | Randomized controlled research design  Two conditions: Experimental Group (exergames combined with physiotherapy), and Control Group (only physiotherapy) | *Wii Fit program* (Aerobics)  *Wii Sport* (Ping pong, Tennis, Boxing, Frisbee)   *Will Balance program* (Training of balance) | Exergames | Console  (Nintendo Wii Fit) | 30 minutes per day, for five days a week | Performance task: endurance task, 2 min walking distance test (2MWY)  Self-report questionnaires: Human Activity Profile (HAP), Functional Assessment of Cancer Therapy Bone Marrow Transplantation (FACT-BMT), Hospital Anxiety and Depression Scale (HADS) | Results showed that muscle strength and physical well-being decreased, while the value of distress increased significantly in both groups. Exergaming on Nintendo Wii resulted in decreased anxiety and depression and improved emotional well-being. The control group showed a contrariwise pattern of these features. |
| Siervo et al [108] | To examine whether behaviors conducted seated (television viewing, video gaming) induce different eating patterns associated with differential levels of the stress response. | 72 participants who were overweight/obese adult males familiar with both FIFA and Call of Duty | 27.3  years old | Randomized controlled research design  Three conditions:  Two Experimental Groups (the Non-violent Video Game Group, and the Violent Video Game Group), and Control Group (non-violent television group) | *FIFA 2013* | Sports video game | Console  (Sony Playstation 3) | 1 hour in one session | Physiological:  heart rate, blood pressure  Self-report questionnaires: VAS stress | Playing video games in overweight/obese adult males is associated with an acute stress response relative to watching non-violent television, associated with greater subsequent food intake. |
|  |  |  |  |  | *Call of Duty* | Action video game (shooter video game) |  |  |  |  |
| Sil et al  [123] | To evaluate the feasibility and efficacy of passive and interactive videogame distraction on behavioral distress for a preschool-aged child receiving repeated burn dressing changes. | A 4-year-old girl who sustained second-and third-degree burns to her shoulders, neck, chest, bilateral forearms, and left tight from an accidental scald injury | 4 year old | Quasi-experimental  Two conditions: Passive Video Games Distraction (watching video games), and Interactive Video Games Distraction (playing video games) | *Go Diego Go! Safari Rescue* | Exergame | Console (Nintendo Wii) | About 40 minutes (during the dressing change) | Behavioral observation scale:  Observation Scale of Behavioral Distress (OSBD)  Self-report questionnaires:  VAS-A Parents and nurse report of child distress | Results revealed significantly lower behavioral distress and greater cooperation during interactive videogame distraction relative to passive videogame distraction. |
| Singh et al [116] | To examine the impact of exergames on psychological well-being, upper limb motor function, and reaction time in adults with physical disabilities. | 18 participants with physical disabilities | 22.7 ± 4.2 years old | Quasi-experimental, pretest-posttest design  One condition (playing exergames) | *Wii Sport* (Tennis, Bowling, Boxing) | Exergames | Console  (Nintendo Wii Fit) | Each game for 10 minutes in one session | Self-report questionnaires: Depressive, Anxiety and Stress Scales Questionnaire (DASS), Capabilities of Upper Extremity Instrument (CUE) | There was a decrease in the anxiety subscale after intervention using exergames. No differences emerged in stress, depression, and Upper limb motor after the intervention. |
| Snodgrass et al [124] | To analyze whether problematic online gaming emerges as a response to life stress. | 133 participants recruited online | 34.6% over 30 years old | Cross-sectional/ correlational research design | *World of Warcraft (WoW)* | Role-play video game (massively multiplayer online role-playing game) | PC | None | Self-report questionnaires: Perceived Stress Scale (PSS), WoW Relaxing Escape Scale, WoW Engagement, Problematic WoW Play Scale | Problematic online gaming in WoW represents a response to pre-existing life stress, which for highly stressed individuals magnifies rather than relieves their suffering. Less stressed individuals manage to play WoW to enhance their offline lives. By contrast, more highly stressed players further magnify the stress and suffering in their lives by playing problematically the online game within which they sought refuge from their offline problems. |
| Viana et al [120] | To evaluate the effects of a single session of the exergame Zumba Fitness on healthy young women's anxiety state. | 40 participants who were healthy women recruited among undergraduate students | 22.9 ± 3.7 years old | Quasi-experimental, pretest-posttest design  One condition (playing exergames) | *Zumba Fitness* | Exergame | Console (Microsoft Xbox 360 with Xbox Kinect) | 20 minutes in one session | Self-report questionnaires: STAI-Y1, Physical Activity Enjoyment Scale (PACES) | A single exergame session significantly reduced the state anxiety in a non-clinical sample of healthy women. However, no statistically significant correlation emerged between enjoyment and absolute or relative change in state anxiety. |
| Watanabe et al [82] | This study investigated Pokémon GO's relationships and psychological distress from an existing workers' cohort in Japan. | 3.915 participants recruited from full-time workers via e-mail and their company website | 42 ± 10 years old | Cross-sectional/ correlational research design | *Pokémon GO* | Augmented reality  game | Mobile (smartphone) | None | Self-report questionnaires: Pokémon GO players, Brief Job Stress Questionnaire (BJSQ), WHO Health and Work Performance Questionnaire (WHO-HPQ) | Improvement in psychological distress was significantly more significant among Pokémon GO players than among non-players. Pokémon GO may help improve psychological distress among workers. |
| Yeh  [121] | To investigate how “out-of-school” video games influence creativity and emotions. | 36 participants who were volunteers with limited game experience | 25.2 ± 3.4 years old | Quasi experimental, pretest-posttest design  Two Conditions: Action Video Game Condition, and Casual Game Condition | *Light Heroes* | Action video game (shooter video game) | PC | 10 minutes each game in one session | Performance task: the idea generation task  Self-report questionnaires:  Self-rating emotion scale on valence, arousal, desire, and stress | Action gameplay elicited higher arousal and stress than casual gameplay. After playing the action game, participants performed higher on originality, elaboration, and flexibility than after playing the casual game, but not productivity. |
|  |  |  |  |  | *Clusterz* | Casual video  game |  |  |  |  |
| Yuen et al [122] | To evaluate a home-based exercise program's effectiveness using the Wii Fit system in patients with systemic lupus erythematosus (SLE). | 15 participants who were sedentary African American women with SLE experiencing moderate to severe fatigue | At least 18 years old | Quasi-experimental, pretest-posttest design  One condition (playing exergames) | *Wii Fit program* (Aerobic, Strengthening) | Exergames | Console  (Nintendo Wii Fit) | 30 minutes for three days a week, for ten weeks | Self-report questionnaires: Fatigue Severity Scale (FSS), Hospital Anxiety and Depression Scale (HADS), Pittsburgh Sleep Quality Index (PSQI), Short-form of the McGill Pain Questionnaire (SF-MPQ) | After the Wii Fit exercise program, participants' anxiety levels and overall intensity of total pain experience significantly reduced. Also, perceived fatigue severity and body weight and waist circumference diminished. |
