# Supplementary material for: Commercial Off-The-Shelf Video Games for Reducing Stress and Anxiety: Systematic Review
Source: JMIR Ment Health. 2021 Aug 16;8(8):e28150. doi: 10.2196/28150 (PMC8406113; doi:10.2196/28150)
Supplement: Multimedia Appendix 2 [file mental_v8i8e28150_app2.docx]

|  | **2. Quantitative randomized controlled trial** | | | | | **3. Quantitative non-randomized** | | | | | **4. Quantitative descriptive** | | | | | **Overall score** |
| --- | --- | --- | --- | --- | --- | --- | --- | --- | --- | --- | --- | --- | --- | --- | --- | --- |
| **Study** | **2.1** | **2.2** | **2.3** | **2.4** | **2.5** | **3.1** | **3.2** | **3.3** | **3.4** | **3.5** | **4.1** | **4.2** | **4.3** | **4.4** | **4.5** |  |
| Alves et al  [109] | No | Yes | Yes | Yes | Yes |  |  |  |  |  |  |  |  |  |  | 4 |
| Bouchard et al [86] | Yes | Yes | Yes | Yes | Yes |  |  |  |  |  |  |  |  |  |  | 5 |
| Butler et al [113] | Yes | Yes | Yes | Can’t tell | Yes |  |  |  |  |  |  |  |  |  |  | 4 |
| De Morais [117] |  |  |  |  |  | Yes | Yes | Yes | No | Yes |  |  |  |  |  | 4 |
| Fish et al [114] | Yes | Yes | Yes | Yes | Yes |  |  |  |  |  |  |  |  |  |  | 5 |
| Fish et al [102] | No | No | Yes | Can’t tell | Yes |  |  |  |  |  |  |  |  |  |  | 1 |
| Horsch et al [115] | Yes | Yes | Yes | Yes | Yes |  |  |  |  |  |  |  |  |  |  | 5 |
| Hua et al [110] | Yes | Yes | Yes | Can’t tell | Yes |  |  |  |  |  |  |  |  |  |  | 4 |
| Huang et al [85] | Yes | Yes | Yes | Yes | Yes |  |  |  |  |  |  |  |  |  |  | 5 |
| Huang et al [111] | No | Yes | Yes | Yes | Yes |  |  |  |  |  |  |  |  |  |  | 4 |
| Jahouh et al [112] | No | Yes | Yes | Yes | Yes |  |  |  |  |  |  |  |  |  |  | 4 |
| Naugle  [118] |  |  |  |  |  | Yes | Yes | Yes | No | Yes |  |  |  |  |  | 4 |
| Özükoç  [103] | No | Can’t tell | Yes | Can’t tell | Yes |  |  |  |  |  |  |  |  |  |  | 2 |
| Pallavicini & Pepe [119] |  |  |  |  |  | Yes | Yes | Yes | No | Yes |  |  |  |  |  | 4 |
| Porter & Goolkasian [106] | No | Yes | Yes | Can’t tell | Yes |  |  |  |  |  |  |  |  |  |  | 2 |
| Roy & Ferguson  [107] | No | Yes | Yes | Can’t tell | Yes |  |  |  |  |  |  |  |  |  |  | 2 |
| Rupp et al  [104] | No | Can’t tell | Yes | Can’t tell | Yes |  |  |  |  |  |  |  |  |  |  | 1 |
| Russoniello et al [36] | No | Can’t tell | Yes | Can’t tell | Yes |  |  |  |  |  |  |  |  |  |  | 1 |
| Shin et al  [80] |  |  |  |  |  | No | Yes | Yes | No | Yes |  |  |  |  |  | 2 |
| Schumacher et al [105] | No | Can’t tell | Yes | Can’t tell | Yes |  |  |  |  |  |  |  |  |  |  | 1 |
| Siervo et al  [108] | No | Yes | Yes | Can’t tell | Yes |  |  |  |  |  |  |  |  |  |  | 2 |
| Sil et al  [123] |  |  |  |  |  |  |  |  |  |  | No | No | Yes | Yes | Yes | 2 |
| Singh et al  [116] |  |  |  |  |  | No | No | No | No | Yes |  |  |  |  |  | 0 |
| Snodgrass et al [124] |  |  |  |  |  |  |  |  |  |  | No | Yes | Yes | Yes | Yes | 4 |
| Viana et al  [120] |  |  |  |  |  | Yes | Yes | Yes | No | Yes |  |  |  |  |  | 4 |
| Watanabe et al [82] |  |  |  |  |  |  |  |  |  |  | Yes | Yes | Yes | Yes | Yes | 5 |
| Yeh  [121] |  |  |  |  |  | Yes | Yes | Yes | No | Yes |  |  |  |  |  | 4 |
| Yuen et al  [122] |  |  |  |  |  | Yes | Yes | Yes | No | Yes |  |  |  |  |  | 4 |
